# Deep Learning for Sensor-Based Sport Performance and Health Monitoring: A Review of Wearable, Vision-Based, and Multimodal Sensing Approaches

**DOI:** 10.3390/s26144384

**Published:** 2026-07-10

**Authors:** Liu Liu, Xinyu Hu, Hong Wei, Ziqian Yang, Tao Sun

**Affiliations:** 1 Department of Physical Education, Jiangsu Maritime Institute, Nanjing 211199, China; 20060305@jmi.edu.cn; 2 College of Furnishings and Industrial Design, Nanjing Forestry University, Nanjing 210037, China; 3 College of Engineering, University of Michigan, Ann Arbor, MI 48109, USA; aureoleh@umich.edu; 4 Nanjing Institute of Agricultural Mechanization, Ministry of Agriculture and Rural Affairs, Nanjing 210014, China

**Keywords:** sport performance analysis, athlete health monitoring, wearable sensors, vision-based sensing, multimodal fusion, deep learning, injury risk prediction, rehabilitation monitoring

## Abstract

Recent advances in wearable, vision-based, trajectory, physiological, and multimodal sensing technologies, together with deep learning, have enabled continuous, objective, and individualized assessment of sport performance and athlete health. Unlike prior reviews that primarily focus on a single sensing modality, sport, or algorithmic series, this review integrates wearable, vision-based, trajectory, physiological, and multimodal sensing streams with deep learning models across both performance analysis and athlete health monitoring, thereby clarifying modality-task-model relationships and translational limitations. This review synthesizes recent progress in sensor-based sports intelligence, focusing on how heterogeneous data streams are transformed into performance- and health-related decision support. The reviewed applications include athlete and ball perception, multi-object tracking, pose estimation, action recognition, trajectory and tactical analysis, training-load and fatigue monitoring, injury-risk prediction, rehabilitation monitoring, and return-to-play support. Deep learning architectures, including CNNs, LSTMs, GRUs, TCNs, Transformers, attention mechanisms, graph neural networks, and multimodal fusion models, are discussed in relation to their suitability for visual, temporal, spatial, physiological, and multisource data. This review further identifies key challenges, including data heterogeneity, annotation scarcity, limited cross-sport and cross-device generalization, real-time deployment constraints, model interpretability, privacy protection, and ethical governance. Moving forward, research efforts should focus on the development of standardized datasets, reliable multimodal data fusion strategies, self-supervised and transfer learning approaches, and deployment on edge or cloud computing platforms. Additionally, enhancing interpretability through explainable AI and implementing closed-loop, individualized monitoring systems are critical. By synthesizing advances in sensing technologies, deep learning methodologies, and real-world applications, this review aims to provide a practical reference for optimizing athletic performance, preventing injuries, guiding rehabilitation, and supporting long-term health management of athletes.

## 1. Introduction

Wearable sensors, vision-based systems, optical trackers, GPS, and physiological monitoring technologies have grown rapidly. These advances are transforming how sport performance and athlete health are analyzed. Previously, evaluations relied mainly on expert observation, experience-based judgment, and post-event statistics. Nowadays, data-driven frameworks increasingly supplement these traditional approaches. Continuous sensing, intelligent modeling, and real-time feedback enable more objective and timely assessments. In high-performance training and competition, variables such as athlete trajectories, posture, acceleration, angular velocity, heart rate, muscle activity, fatigue status, and rehabilitation progress can now be recorded with high continuity and granularity. Recent sports medicine studies have shown that wearable sensing technologies are increasingly used in functional assessment, injury monitoring, postoperative rehabilitation, and training feedback, thereby providing a more objective, quantifiable, and traceable basis for sports science and clinical decision-making [1].

Sensor data in sports settings can be broadly classified into vision/camera-based data, trajectory and positioning data, wearable inertial data, and physiological or biosignal data. These modalities capture movement appearance, spatial evolution, local movement dynamics, and internal physiological states, respectively. Studies on motion capture and sensing technologies in sports have emphasized that systems differ substantially in accuracy, cost, portability, real-time capability, and use cases; consequently, sensor selection should be guided by the sport, research question, and practical context [2].

To maintain a coherent scope, this review treats sport performance analysis and athlete health monitoring as related but distinct endpoints. Performance-oriented applications emphasize perception, trajectory reconstruction, action quality assessment, and tactical decision support, whereas health-oriented applications emphasize internal load, fatigue, injury risk, rehabilitation, and return-to-play decisions. This distinction is used throughout the manuscript to reduce repetition and to connect each sensing modality with its most appropriate analytical tasks.

Deep learning has substantially advanced the analysis of visual/video-based sports data. In football, for example, SoccerNet-v2 provides a large-scale benchmark for broadcast video understanding, covering tasks such as action spotting, camera-shot understanding, and replay grounding, thereby extending sports video analysis beyond isolated event recognition toward more comprehensive match understanding [3]. SportsMOT and related multi-object tracking datasets further address challenges such as high-speed motion, visually similar players, and frequent occlusion in basketball, volleyball, and football, making player detection, identity preservation, and trajectory reconstruction foundational tasks in intelligent sports analysis [4]. These studies suggest that visual sensing has moved beyond simple player and ball recognition. In many sports scenarios, it is also used to describe actions, movement patterns, and interactions among players. For performance analysis, deep learning is therefore no longer limited to basic perception tasks. It is increasingly applied to spatiotemporal modelling, game-state interpretation, and decision support. For example, object detection, trajectory estimation, and graph convolutional networks have been combined to analyze player–ball interactions in real time. Such methods can also estimate distance, speed, and interaction patterns among athletes [5]. TacticAI provides another example. It uses geometric deep learning to support tactical interpretation and decision-making in team sports [6].

Health-related applications form another important branch of sensor-driven sports intelligence. Unlike tactical analysis, health monitoring is more closely related to load response, fatigue, injury risk, and recovery status. These outcomes are affected by both external workload and internal physiological changes. Previous injury history, functional capacity, and sport-specific demands also need to be considered. When movement data, physiological signals, and temporal information are analyzed together, models can describe athlete status from multiple perspectives. This is why multimodal sensing is increasingly used to improve recognition robustness by integrating complementary movement information [7].

Deep learning is increasingly applied to real-time health prediction using data from wearable sensors. In many studies, recurrent neural networks have analyzed athlete health signals collected from these devices. These approaches demonstrate the feasibility of continuous state recognition and real-time monitoring of physiological status [8].

Injury-risk prediction is another important application. Longitudinal studies of professional football players show that combining internal and external load measures with machine learning models can estimate muscle-injury risk. Such models also help clarify the influence of training and match load on injury occurrence [9]. Additionally, biomechanical testing combined with explainable machine learning has identified features such as hamstring strength, muscle stiffness, and bilateral asymmetry. These findings highlight the need for models that are both accurate and interpretable [10].

Rehabilitation monitoring and return-to-sport decision support form another key application area. Traditional rehabilitation assessment often relies on periodic physical tests, clinical experience, or fixed thresholds. These methods may not fully capture recovery quality under real movement conditions. Recent work has leveraged early post-operative physical performance measures after anterior cruciate ligament reconstruction to train machine learning models for predicting return-to-sport outcomes 12 months later [11]. This research indicates that integrating wearable sensors, functional testing, and intelligent modeling can shift assessment from checking if an athlete meets a fixed threshold toward evaluating whether they can return to sport safely and effectively.

Despite these advances, sensor-based sport performance analysis and health monitoring still face several barriers. Sports sensor data are strongly affected by context. The type of sport, acquisition device, camera viewpoint, sensor placement, and athlete level can all change the data distribution. This makes it difficult for models trained in one setting to perform reliably in another. The shortage of high-quality labeled data is another major limitation. This problem is particularly evident in fatigue recognition, injury-risk prediction, and rehabilitation-outcome modeling. In these tasks, sample sizes are often small, target events are rare, and variable definitions differ across studies. Scoping reviews on sports injury prediction have also reported similar concerns. Although machine learning methods are increasingly used, their practical and clinical translation is still limited by small datasets, inconsistent injury definitions, and insufficient external validation [12].

Sports applications also require fast and efficient model deployment. During training or competition, feedback often needs to be delivered with low latency. However, deep learning models with high accuracy may require considerable computational resources. Internet of Things platforms, edge computing, and deep learning have therefore been introduced into real-time athlete monitoring systems. These technologies can improve processing efficiency, feedback speed, and field deployment [13].

In practical settings, accuracy alone is not sufficient. Models also need to be robust, interpretable, privacy-preserving, and compatible with human decision-making. Recent studies on deep learning for sport performance analysis have therefore emphasized multimodal fusion, real-time analytics, and cross-sport adaptability. These aspects are essential for improving the practical value of intelligent systems in training and competition environments [14]. Accordingly, this review examines recent progress in sensor-driven sports intelligence, focusing on sport performance enhancement and athlete health monitoring. Unlike reviews centered on a single algorithm, sport, or sensing device, this paper emphasizes the relationships among sensing modalities, data structures, deep learning models, and application tasks. The main objectives of this review are to:

(1) Summarize the major types of sensor data used in sports and their roles in performance analysis and health monitoring;
(2) Review deep learning applications in athlete and ball perception, action recognition, trajectory modeling, tactical analysis, training-load assessment, injury-risk prediction, and rehabilitation monitoring;
(3) Analyze key challenges related to data quality, cross-scenario generalization, real-time deployment, model interpretability, and practical translation;
(4) Discuss future directions, including multimodal fusion, edge intelligence, trustworthy modeling, and individualized closed-loop feedback.

The remainder of this paper is organized around sensor-data foundations, deep learning methods, representative application scenarios, key challenges, and future research directions, with the aim of providing a structured reference for sensor-driven sports intelligence and athlete health management. Section 3, Section 4 and Section 5, therefore, follow a consistent sequence from sensor modality and data structure to model series, application objective, and limitation, while Section 6 synthesizes cross-cutting challenges and future directions.

## 2. Review Scope and Literature Search Strategy

This review provides a structured narrative on deep learning approaches for analyzing sensor-based sport performance and monitoring athlete health. Its goal is to clarify how sensing modalities, data types, model architectures, application tasks, and practical challenges interrelate.

To improve transparency and reproducibility, the review process was organized into four steps: database search, relevance screening, thematic classification, and narrative synthesis. The search design and thematic categories were informed by recent reviews and scoping studies on wearable sports medicine, motion-capture technologies, injury-risk prediction, and deep learning applications in sport performance analysis [1,2,12,14].

Relevant studies were collected through searches of Web of Science Core Collection, Scopus, PubMed, IEEE Xplore, and Google Scholar. The focus was on literature published between 2019 and 2026. Older studies were included when they introduced widely used datasets, foundational architectures, or influential methods.

Search terms combined concepts from sports applications, sensing technologies, and computational approaches. Examples include “sport performance analysis,” “athlete health monitoring,” “wearable sensor,” “inertial measurement unit,” “physiological sensing,” “computer vision,” “player tracking,” “multimodal sensing,” “deep learning,” “injury risk prediction,” “fatigue monitoring,” and “rehabilitation monitoring.” Reference lists of selected reviews and representative studies were also checked for additional relevant publications.

Studies were deemed relevant if they analyzed sensor-derived sports data and applied deep learning or related machine learning methods to at least one performance- or health-related task. Priority was given to peer-reviewed journal articles, conference papers, benchmark datasets, and recent reviews offering methodological, empirical, or application-specific evidence. Publications outside sports contexts, lacking clear sensing or analysis components, or with insufficient methodological detail, were not emphasized.

Identified studies were classified based on sensing modality, data representation, model architecture, and application objective. They were synthesized to highlight representative technical developments and recurring challenges across studies.

This classification created a common analytical pathway from sensing source to data representation, model series, application outcome, and limitation.

## 3. Sensor Modalities and Data Characteristics in Sports

Accurate perception of athlete movement is fundamental to sport performance analysis and health monitoring. Compared with general human activity recognition, sports scenarios involve distinctive challenges, including high-speed movement, intense physical interaction, complex occlusion, pronounced individual differences, and well-defined performance objectives. Consequently, sensor data not only serve as model inputs but also influence task definition, model architecture, and practical application.

In sports contexts, primary sensing modalities include vision/camera-based data, trajectory and positioning data, wearable inertial data, physiological and biosignal data, and multimodal fused data. These data streams provide complementary evidence for deep learning models to perceive, interpret, assess, and support decision-making.

Different modalities provide complementary advantages and constraints. Vision data offer rich semantic information but are sensitive to occlusion, lighting, and camera viewpoint. Trajectory and positioning data enable structured spatial analysis but depend on accurate localization and stable identity tracking. Wearable inertial sensors support continuous movement monitoring but can be affected by placement and drift. Physiological signals reflect internal load but are susceptible to noise and inter-individual variability. Prior studies indicate that sensing technology should be selected according to task requirements, including accuracy, real-time capability, cost, and applicability [2].

**Figure 1:**
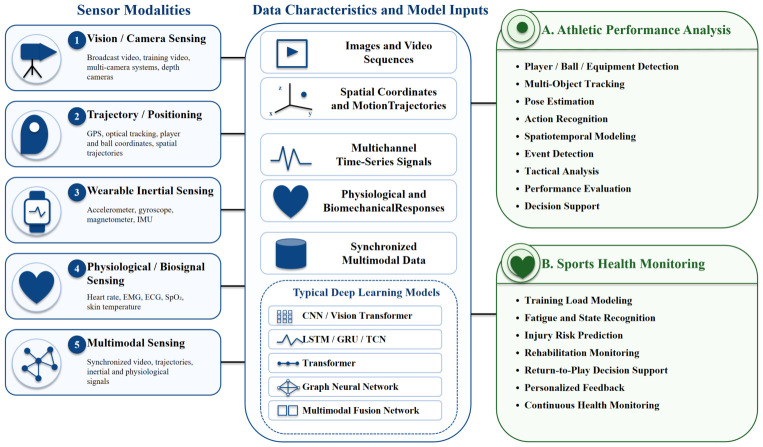
Overview of major sensor modalities, data streams, and their mapping to typical sports applications.

From a modeling perspective, data modality shapes model selection. Image and video data are typically analyzed using convolutional neural networks, video Transformers, or temporal attention models. Trajectory and positioning data are often modeled as time series or dynamic graphs. Inertial and physiological data are represented as multichannel time series and are suitable for one-dimensional convolutions, recurrent neural networks, and temporal Transformers. Multimodal data introduce additional challenges, including temporal synchronization, spatial alignment, feature fusion, and decision-level integration. Studies on sports video action recognition have shown that athletic actions are characterized by high speed, fine-grained categories, multi-agent interactions, and high annotation cost; therefore, models should incorporate both visual features and temporal context. Figure 1 provides an overview of the primary sensor modalities, data types, and their mapping to typical sports applications.

Because each sensor modality imposes distinct constraints on representation learning, Table 1 summarizes the relationships among data structure, model choice, application objective, and limitation. The table complements Figure 1 and shows why modality selection should be task-driven: external behavior and tactical context are better captured by vision and trajectory data, whereas continuous load and health-state monitoring often require inertial and physiological signals. Multimodal designs are needed when target outcomes depend on both movement execution and internal response.

Building on this framework, the following sections examine the characteristics, applications, and modeling constraints of each sensor category.

### 3.1. Vision and Camera-Based Data

Vision and camera-based data are among the most widely used and information-rich sensor modalities in sports intelligence. They are obtained from broadcast videos, training recordings, fixed-camera systems, multi-view setups, depth cameras, and mobile devices. Their advantages include non-contact acquisition and strong scene-reconstruction capacity, allowing simultaneous capture of athlete movement, equipment status, venue context, and team interaction. Accordingly, vision-based sensing supports sport perception and movement understanding.

The development of large-scale sports video datasets has substantially advanced deep learning-based visual analysis. For example, SoccerNet-v2 expands annotated football videos to provide benchmarks for action spotting, shot understanding, and replay alignment, enabling analyses that move beyond isolated action detection toward full-match context understanding [3]. These datasets emphasize not only whether an action occurs but also its temporal location, camera composition, and relationship to key match events. For performance analysis, this supports modeling from short action segments to extended sequences representing the entire match.

For technical and individual sports, vision data support fine-grained analysis of action structure. The FineGym dataset for gymnastics provides hierarchical annotations that divide complete routines into actions and sub-actions, enabling detailed action understanding [15]. Many technical movements consist of sequential phases, such as takeoff, flight, rotation, support, and landing, requiring models to capture stage-specific dynamics. Phase-based action modeling improves the discrimination of subtle differences and supports skill evaluation and training feedback.

**Figure 2:**
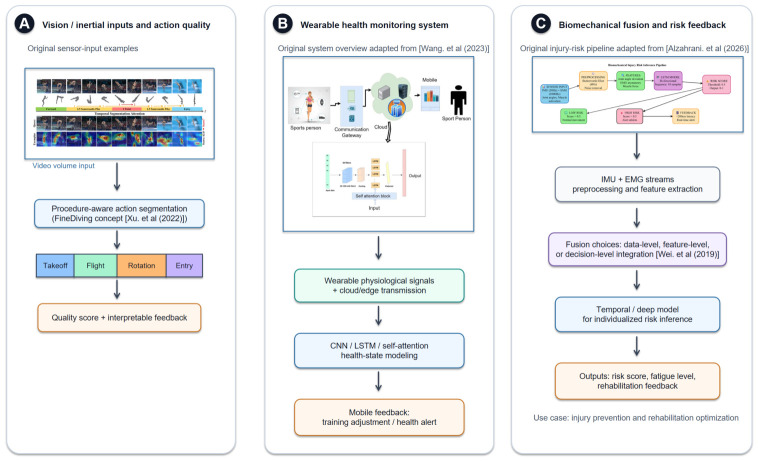
Hybrid sensor-data processing pathways. (**A**) Vision and inertial input representations combined with a procedure-aware action quality assessment workflow based on FineDiving [16]. (**B**) Wearable/cloud health-monitoring system and CNN-LSTM-self-attention processing adapted from [23]. (**C**) Wearable biomechanics injury-risk pipeline adapted from [24], integrated with common multimodal fusion choices for downstream risk alerts and rehabilitation feedback [7,24].

Movement-quality assessment is another major application of vision data in performance analysis. Whereas action recognition identifies what was performed, quality assessment evaluates how it was performed. The FineDiving dataset for diving includes action sequences with scoring information, emphasizing the importance of temporal structure for score prediction and model interpretability [16]. This is particularly relevant in sports where execution quality, rhythm control, posture, and phase transitions are more important than action categories alone, such as gymnastics, diving, figure skating, martial arts, and other technical skills. Figure 2A schematically illustrates this procedure-aware pipeline, in which a diving video is decomposed into action phases before temporal modeling produces a quality score and interpretable phase-level feedback.

In practical applications, vision data generates not only image- or video-level classifications but also intermediate representations. Athlete bounding boxes support multi-object tracking and trajectory reconstruction; keypoints can form skeleton sequences for action recognition and skill assessment; and player and ball positions can support interaction modeling and tactical analysis. Nevertheless, sports vision data face challenges such as high-speed motion, motion blur, multiple occlusions, similar uniforms, camera switching, and sport-specific variation. Occlusion handling, dynamic backgrounds, computational efficiency, and cross-sport generalization are identified as persistent challenges in sports pose estimation and tracking [17].

With advances in high-resolution video acquisition, multi-view synchronization, markerless pose estimation, and video-based deep models, vision data are expected to remain central to sports intelligence.

### 3.2. Trajectory, Positioning, and Optical Tracking Data

Trajectory, positioning, and optical tracking data are essential for capturing spatial behavior and team interaction in sports. Unlike vision-based data, which directly record visual appearance, trajectory and positioning data are typically structured as spatial coordinates, velocities, accelerations, motion directions, distances, and identity labels, providing a direct representation of athletes, balls, or equipment over time and space. These data are obtained from global positioning systems, local positioning systems, optical tracking setups, multi-camera tracking systems, or coordinate data generated from video-based detection and multi-object tracking algorithms. With their widespread deployment in professional leagues and high-level training, high-frequency spatiotemporal tracking data have become a key foundation for performance analysis, tactical modeling, and load assessment [18].

Trajectory and positioning data are highly structured and are often represented as continuous time series, in which each timestamp encodes the 2D or 3D coordinates of athletes or objects. Derived metrics include speed, acceleration, distance covered, sprint counts, spatial occupancy, formation width, offensive-defensive spacing, and inter-player distance. Compared with raw video, trajectory data offer advantages in privacy protection, computational efficiency, and tactical analysis because models can directly process positional and movement relations without extracting objects from pixels. In team sports, these data are particularly suitable for analyzing formation changes, coordinated movement, defensive pressure, passing options, and space creation, making them widely applicable to football, basketball, rugby, ice hockey, and field hockey.

In deep learning applications, trajectory and positioning data can be modeled as time series, spatial point sets, or dynamic graph structures. For individual athletes, trajectory sequences support future-movement prediction, pattern recognition, and workload assessment. In team contexts, the positions of multiple athletes and the ball can form dynamic interaction networks, where nodes represent players or objects and edges encode distance, direction, passing relations, or defensive pressure. Using such representations, recurrent neural networks, temporal convolutional networks, Transformers, and graph neural networks can learn trajectory-evolution patterns and team-coordination behaviors. High-resolution spatiotemporal tracking has transformed sports statistics, performance prediction, tactical-pattern recognition, and spatial-value evaluation [18].

Trajectory data can be obtained from dedicated positioning devices or automatically generated through optical tracking and video analysis. For example, SoccerNet-Tracking provides multi-object tracking annotations for football, including players, referees, and the ball, across both short sequences and long-duration videos, serving as a benchmark for trajectory extraction, identity maintenance, and long-term tracking [19]. SportsMOT extends this benchmark setting to basketball, volleyball, and football, highlighting challenges such as high-speed motion, acceleration changes, and similar appearances. These findings indicate that stable trajectory recovery from video remains a key challenge in sports intelligence [4]. Trajectory and positioning data also provide natural inputs for graph-based tactical modeling because team tactics emerge from evolving spatial relations among multiple entities. For instance, TacticAI shows how positional and geometric information can support higher-level tactical analysis and decision-making [6].

Nevertheless, trajectory, positioning, and optical tracking data also have limitations. Systems differ in sampling rate, spatial precision, field adaptability, and cost, which affect cross-study comparability. Video-derived trajectories are vulnerable to occlusion, camera switching, rapid small-object motion, and identity confusion, particularly in long-term ball tracking. Although structured, trajectory data generally lack fine-grained action details, body posture, and physiological responses, limiting their ability to interpret technical performance or internal load when used alone. Commercial and privacy constraints on professional datasets further restrict public data availability and generalization studies.

Trajectory, positioning, and optical tracking data provide structured spatiotemporal representations for object tracking, tactical understanding, performance evaluation, and decision support. Advances in high-precision positioning, automated video tracking, multimodal synchronization, and graph-based deep learning will further expand their practical use.

### 3.3. Wearable Inertial Sensor Data

Wearable inertial sensor data are among the most widely used modalities in sport performance analysis and athlete health monitoring. These data are primarily collected using inertial measurement units (IMUs), which integrate accelerometers, gyroscopes, and magnetometers. Compared with vision-based sensing, wearable inertial sensors are compact, cost-effective, suitable for long-term use, and unconstrained by camera viewpoint, allowing continuous recording of local body movement during training, competition, and rehabilitation. Typical measurements include tri-axial acceleration, angular velocity, orientation angles, impact load, step frequency, movement rhythm, and segmental motion, which can be used to characterize exercise intensity, technical skills, training load, and functional recovery.

In sport performance analysis, wearable inertial sensors complement traditional training logs and observational assessments. For instance, studies on RaceRunning athletes have used waist and lower-limb sensors to continuously capture training load, impact direction, and running efficiency, demonstrating that wearable systems provide more detailed feedback than simple measures of duration, distance, or repetitions. They also allow adaptations in technique during repeated sprints to be detected, indicating that inertial data reflect not only how much athletes train but also how they train, thereby supporting individualized training adjustment [20].

From a deep learning perspective, inertial data are typically represented as multichannel time series suitable for one-dimensional convolutional networks, recurrent neural networks, temporal convolutional networks, and Transformer-based models. These approaches can extract cyclical patterns, impact characteristics, rhythm variations, and localized motion features from continuous acceleration and angular velocity signals, enabling applications in action recognition, exercise-intensity estimation, fatigue assessment, and anomaly detection. Recent work on oxygen-consumption estimation in team sports combined IMU, heart-rate, and respiratory data with machine learning models, comparing LSTM, CNN, MLP, and XGBoost approaches. The results indicated that LSTM models trained on raw IMU data provided accurate VO2 predictions, confirming that inertial sensors can support non-invasive internal-load estimation [21].

Nevertheless, wearable inertial data have limitations. Sensor placement, attachment method, and individual movement habits can significantly affect signal quality, reducing cross-study comparability. IMUs are also susceptible to drift, noise, and impact artifacts, which necessitate filtering, calibration, and standardization. Moreover, a single sensor typically captures only local segmental motion, limiting its ability to represent full-body movement and overall motion context. Consequently, inertial data are often combined with vision, trajectory, or physiological signals to improve interpretability in performance and health assessment.

Wearable inertial sensors provide portable, continuous, and high-temporal-resolution motion measurements, serving as a fundamental data source for training-load monitoring, action analysis, fatigue detection, and rehabilitation evaluation in sports contexts.

### 3.4. Physiological and Biosignal Data

Physiological and biosignal data are primarily used to capture athletes’ internal load and physiological responses during training, competition, and rehabilitation, providing critical information for health monitoring and individualized training adjustment. Common signals include heart rate, heart-rate variability, electrocardiography (ECG), electromyography (EMG), blood oxygen saturation, skin temperature, respiration rate, sweat-related measures, and localized muscle activity. Unlike vision, trajectory, or inertial data, which reflect external behavior, physiological signals characterize internal states and provide insight into fatigue accumulation, recovery status, cardiorespiratory load, muscle activation, and potential health risks.

In practice, physiological data are often collected using wearable devices and mobile platforms to enable continuous health monitoring. For example, real-time wearable systems designed for athlete training have used strain gauges, temperature sensors, and pulse sensors to measure muscle contraction, body temperature, and pulse rate, providing immediate feedback for athletes and coaches during training sessions [22]. In addition, cloud-enabled health monitoring systems integrate physiological and motion data, including heart rate, acceleration, and body temperature, with deep learning models such as CNNs, LSTMs, and self-attention mechanisms to predict athlete health status [23]. Typical workflows include data acquisition, wireless transmission, cloud or edge processing, deep learning modeling, and mobile feedback (Figure 2B).

This framework highlights that physiological signals derive value not only from single-point measurements but also from integration with communication, computation, and intelligent modeling, enabling continuous monitoring for training regulation and health alerts. Figure 2B further clarifies how captured physiological and motion signals are transmitted, stored, processed by deep learning models, and translated into health feedback.

From a modeling perspective, physiological signals exhibit temporal dependencies and inter-individual variability. Changes in heart rate, EMG, or respiration are influenced not only by exercise intensity but also by training level, recovery state, environmental conditions, and psychological stress. Consequently, deep learning models must capture temporal trends, abnormal fluctuations, and individualized response patterns. Research using IMU-based wearable devices and recurrent neural networks has shown that continuous physiological data can predict football players’ health status and provide a foundation for real-time monitoring [8]. When combined with external load, movement features, and exercise intensity, these models offer a more comprehensive representation of the relationship between internal physiological states and observable performance.

Despite their value, physiological and biosignal data present several challenges. Signals can be affected by motion artifacts, sweating, skin contact, sensor-attachment stability, and environmental temperature. Individual baseline differences and variable load responses make it difficult to apply uniform thresholds. Moreover, the relationship between physiological signals and performance outcomes or injury risk is often nonlinear and context-dependent. Therefore, physiological signals are most effective as a complementary modality alongside inertial, trajectory, and vision data, supporting fatigue detection, load management, injury-risk assessment, and individualized feedback in sports health monitoring.

### 3.5. Multimodal Sensor Fusion Data

Multimodal sensor fusion refers to the synchronous acquisition and integration of multiple sensing streams within the same training, competition, or rehabilitation context. Its purpose is to characterize athletic movement and physiological status from complementary perspectives. By integrating vision, trajectory, inertial, and physiological sources, multimodal fusion can compensate for the limitations of individual modalities and improve model robustness in complex sports environments.

Within deep learning frameworks, multimodal fusion is commonly implemented at the data, feature, or decision level. Data-level fusion synchronizes and aligns different modalities before model input. Feature-level fusion extracts modality-specific representations and then combines them through concatenation, attention weighting, or projection into a shared latent space. Decision-level fusion integrates predictions from independently trained modality-specific models. For example, in video-inertial fusion, researchers have processed camera-based video sequences and wearable inertial signals using 3D and 2D convolutional networks, respectively, and compared feature-level and decision-level fusion strategies. The fused models outperformed unimodal baselines, indicating the complementary value of visual and inertial information for action recognition [7].

Multimodal fusion is particularly important in sports health monitoring because athlete status depends on external workload, movement patterns, physiological responses, and recovery capacity. Recent work has combined IMU and surface EMG signals for real-time biomechanical risk assessment and rehabilitation tracking [24]. In team sports, the joint use of IMU, heart-rate, and respiratory signals has also improved estimation of internal physiological demand [21]. Figure 2C summarizes the typical fusion pathway, including preprocessing and synchronization, data-level fusion, feature-level fusion, decision-level fusion, and downstream outputs such as action recognition, injury-risk alerts, and rehabilitation feedback.

Nevertheless, multimodal fusion introduces additional technical challenges. Different modalities vary in sampling frequency, temporal delay, spatial coordinate system, noise characteristics, and missing-data patterns. Reliable synchronization and alignment are therefore prerequisites for effective fusion. Multimodal models also tend to increase computational complexity, which may hinder real-time deployment. In practical settings, some modalities may be unavailable because of sensor failure, occlusion, or unstable attachment, requiring models to handle missing or degraded inputs. Overall, multimodal fusion represents a transition from single-indicator monitoring to comprehensive state understanding. Its value lies in integrating observed movement, measured workload, and physiological response within a unified analytical framework.

### 3.6. Relationships Among Sensor Modalities, Task Objectives, and Model Architectures

Sensor modalities, task objectives, and model architectures are closely interdependent in sports applications. Wearable inertial data and physiological signals are more directly linked to continuous state monitoring and health-risk assessment. Inertial time series support action-cycle detection, training-load estimation, impact analysis, and rehabilitation movement assessment. Physiological and biosignal data support internal-load estimation, fatigue recognition, recovery monitoring, and health-state prediction. Wearable-based health monitoring systems using cloud processing and deep learning have shown that CNN, LSTM, and attention mechanisms can jointly exploit local signal features and temporal dependencies for athlete health-state modeling [23].

Multimodal data further expand both task scope and model design space. Vision-inertial fusion can improve action recognition and pose understanding; trajectory-physiology-load fusion can model the relationship between external performance and internal response; and vision-trajectory-graph fusion can support tactical analysis and game-state understanding. Real-time performance monitoring studies have also shown that wearable sensors, Internet of Things transmission, and deep learning models such as TCN, BiLSTM, and attention mechanisms can support continuous workflows from data acquisition to real-time feedback [25].

Accordingly, sensor modality, task objective, and model architecture should not be treated as independent components. Future method design should emphasize alignment among data, model structure, and application task, rather than pursuing model complexity or isolated performance metrics alone.

## 4. Deep Learning Methods for Sport Performance Analysis

In sport performance analysis, sensor data derive value when transformed into interpretable, measurable, and decision-relevant information through deep learning models. Compared with manual statistics and expert observation, deep learning can learn representations from images, videos, trajectories, poses, and multimodal data. This section reviews how representative tasks progress from low-level perception to higher-level performance, interpretation, and decision support.

At the task level, athlete, ball, and equipment detection provide the basis for sport perception. Multi-object tracking and trajectory reconstruction generate continuous spatiotemporal information, whereas pose estimation and action recognition support the interpretation of technical movements. Spatiotemporal modeling, event detection, and graph neural networks further support tactical analysis, performance assessment, training feedback, and competition decision-making.

Table 2 summarizes the methods reviewed in Section 4 by task, input, model series, output, and limitation. This organization distinguishes low-level perception tasks from higher-level tactical and decision-support tasks.

### 4.1. Athlete, Ball, and Equipment Detection

Athlete, ball, and equipment detection is a fundamental task in sports vision analysis. The goal is to locate and identify sport-related objects in images or video frames, providing inputs for tracking, trajectory reconstruction, pose estimation, action recognition, and tactical analysis. Compared with general object detection, sports detection is more dynamic and domain-dependent because athletes move rapidly, occlude one another, and interact with small, fast-moving objects or sport-specific equipment.

Current sports object detection methods are largely based on deep convolutional networks and one-stage detection frameworks. One-stage detectors, represented by the YOLO series models, offer fast inference and are well-suited to real-time analysis during training and competition. Two-stage detectors may provide higher localization accuracy in some cases, but their computational cost often requires compression or deployment optimization for practical use. The continued evolution of YOLO-based models has improved detection accuracy, inference speed, and deployment feasibility, making them widely used for athlete detection, ball detection, and sport-object recognition [26], which provides a favorable balance between accuracy and real-time efficiency [27].

In sport-specific applications, detection models have expanded from single-object localization to multitask perception. DeepSportLab, for example, provides a unified framework for team-sport analysis by jointly addressing ball detection, player instance segmentation, and pose estimation, illustrating the close relationship among detection, segmentation, pose analysis, and interaction modeling in sports vision [28]. In technical sports such as gymnastics, SCB-YOLOv5 improves YOLOv5 using a lightweight backbone and attention mechanisms to detect standardized athletic movements, demonstrating the potential of object detection in sports instruction and technical correction [29]. In football, YOLOv7 combined with semi-supervised strategies has been used for ball detection and tracking to address small-object size, high-speed motion, and annotation cost [30].

The practical value of athlete, ball, and equipment detection lies mainly in the intermediate representations it provides. Athlete bounding boxes can support identity association; ball positions can support player–ball interaction analysis and event recognition; and detected equipment or field regions can assist action-phase segmentation and spatial normalization.

Sports object detection still faces persistent challenges, including similar player appearances, occlusion, small objects, motion blur, cross-sport variation, and real-time inference constraints. Future work should combine lightweight architectures, small-object enhancement, temporal information, and multitask learning to improve stability and deployability.

### 4.2. Multi-Object Tracking and Trajectory Reconstruction

Multi-object tracking and trajectory reconstruction are key steps in shifting sport performance analysis from frame-level perception to continuous process understanding. Object detection identifies athletes, balls, or equipment at a given moment, whereas performance analysis requires movement paths, identity continuity, velocity changes, and spatial relationships over time.

In team sports, multi-object tracking is shaped by sport-specific characteristics. Football, for example, involves many players with similar uniforms, frequent body occlusion, camera zooming, viewpoint changes, and small distant targets in broadcast footage. The stable player detection and tracking are foundational for subsequent two-dimensional pitch mapping, spatial analysis, and match understanding [31]. Sports-specific tracking therefore cannot rely solely on generic visual association; it should also incorporate field geometry, game semantics, and domain constraints.

Recent studies have adapted tracking methods to the particular challenges of sports video. MV-Soccer proposes a football-oriented framework combining detection, instance segmentation, and tracking with motion vectors and frame-difference information, improving foreground-background separation and tracking performance under occlusion and blurred boundaries [32]. SportSORT addresses appearance similarity, long-term occlusion, and re-entry after leaving the field of view by incorporating domain cues such as jersey color and number, together with correction matching and re-association mechanisms [33]. These studies suggest that sports tracking is moving from generic multi-object tracking frameworks toward specialized models that exploit sport-specific visual and contextual information.

**Figure 3:**
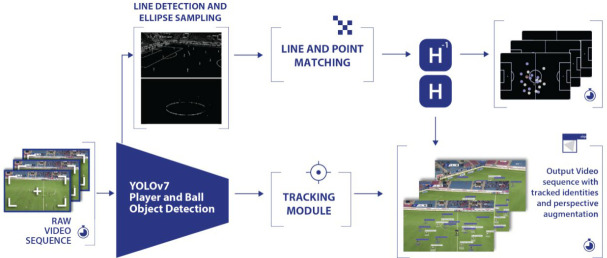
Multi-object tracking and pitch localization workflow in football videos based on FootyVision [34].

Trajectory reconstruction further structures tracking outputs into spatially meaningful data. By combining bounding boxes, identity labels, timestamps, and camera calibration, pixel coordinates in video can be transformed into field coordinates, enabling the calculation of speed, acceleration, distance covered, spatial occupation, inter-player distance, and formation dynamics. FootyVision provides an integrated framework for football videos that combines player and ball detection, tracking, localization, and augmented visualization. It uses YOLOv7 for object detection, integrates feature embeddings, bounding-box overlap, distance, velocity, and Hungarian matching for identity association, and applies field geometry for perspective transformation from video view to pitch coordinates [34]. The workflow shown in Figure 3 illustrates a typical pipeline from raw video input to object detection, multi-object tracking, pitch localization, and augmented spatial analysis. This work highlights that trajectory reconstruction is not limited to obtaining movement paths; rather, it converts visual detections into spatial data that support match understanding and tactical analysis.

Stable trajectory data enable higher-level sport performance analysis. In football, player and ball trajectories can be used to study possession progression, off-ball movement, defensive pressure, and player–ball interactions. In basketball, trajectories can support the recognition of screens, defensive switches, drives, and spacing. In volleyball and tennis, ball trajectories and player positions help interpret hit timing, movement choices, and technical execution. Recent work combining object detection, player tracking, and graph convolutional networks has enabled real-time analysis of player–ball interactions in football, including measures of inter-player distance, player–ball proximity, speed, and total distance covered, demonstrating the value of trajectory data for real-time match insight and tactical support [5].

Despite these advances, multi-object tracking and trajectory reconstruction remain challenging in sports environments. Occlusion, camera switching, distant targets, and high-speed ball motion can lead to missed detections, fragmented trajectories, and identity switches. Because trajectory reconstruction depends on detection quality, camera calibration, and coordinate mapping, front-end errors may propagate into tactical analysis and performance evaluation.

### 4.3. Pose Estimation and Action Recognition

Pose estimation and action recognition represent a transition from object-level perception to semantic understanding of athletic movement. Detection and tracking determine where athletes are and how they move, whereas pose estimation focuses on body keypoints, posture, and joint relationships. Action recognition then maps pose, video, or multimodal representations to technical skills, matching behaviors, or action phases.

Deep learning-based pose estimation in sports can be broadly divided into two-dimensional and three-dimensional approaches. Two-dimensional pose estimation extracts human joint coordinates from images or videos and is commonly used for skill observation, action-phase segmentation, and training feedback. Three-dimensional pose estimation further recovers joint positions and orientations in space, making it more suitable for analyzing movement amplitude, postural control, and biomechanical characteristics. Its applications mainly include motor-skill analysis, action recognition, enhanced coaching tools, and judging support, indicating that pose estimation has evolved from a general computer vision task into a practical tool for sports training and performance analysis [35].

**Figure 4:**
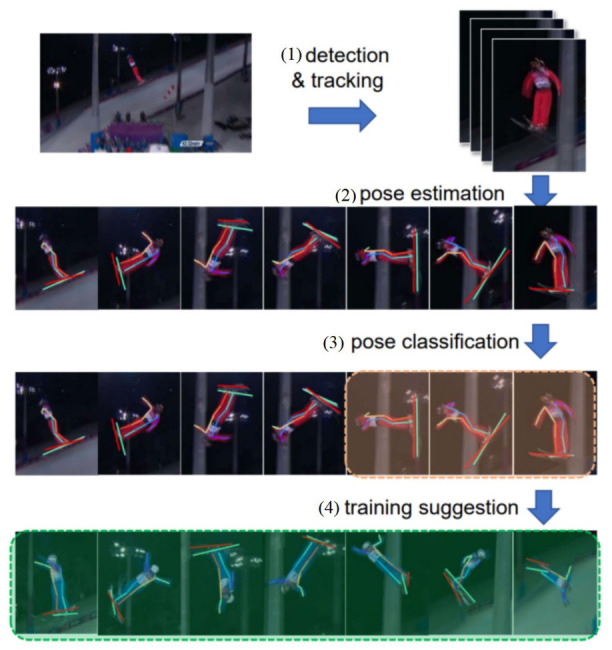
Pose-estimation-based workflow for personalized athletic training assistance [36].

In training assistance and technical feedback, pose estimation provides fine-grained information about movement execution. The AI Coach system, for example, uses human pose estimation from video together with single-person trajectory extraction, joint-relation modeling, and posture correction to provide movement scores, abnormal-posture detection, and visual improvement suggestions [36]. The value of pose estimation lies not only in generating keypoint coordinates but also in translating body-posture changes into interpretable feedback. For movements such as squats, swings, jumps, throws, running, gymnastics, and martial arts, pose-sequence analysis can help identify abnormal joint angles, unstable rhythm, bilateral asymmetry, and missing technical components. Figure 4 illustrates a typical pose-estimation-based workflow for personalized training assistance, in which athletes are detected and tracked, pose sequences are extracted and classified, and training recommendations are generated.

Action recognition extends pose and video representations to specific action categories or movement phases. In team sports, it is commonly used to identify passing, shooting, defending, intercepting, fouling, driving, and tackling behaviors. In individual technical sports, action recognition often focuses on complete skills and their internal phase structure. This field has moved from action classification toward temporal action localization and spatiotemporal localization; models must determine not only what action occurred but also when and where it occurred [37]. This shift indicates that sports action recognition is moving beyond static label classification toward richer spatiotemporal semantic understanding.

From a modeling perspective, sports action recognition commonly uses RGB video, optical flow, skeleton sequences, or multimodal inputs. RGB video retains scene context and appearance information but is sensitive to background, clothing, and camera viewpoint. Skeleton sequences emphasize body structure and motion trajectories, offering stronger privacy protection and clearer action representation. Combining RGB and pose information can therefore exploit both scene semantics and human movement structure. In figure skating fall classification, the Gate–Shift–Pose method integrates skeleton information with RGB images and compares early and late fusion strategies. The findings indicate that adding pose information improves recognition performance, highlighting the importance of skeleton features for capturing complex athletic movement patterns [38].

Pose estimation and action recognition also support movement-quality assessment, which considers both what action was performed and how well it was performed. Compared with standard action classification, quality assessment must consider execution completeness, phase transitions, body posture, rhythm control, and scoring criteria. Recent reviews of vision-based action quality assessment indicate that deep learning and motion capture technologies have accelerated this field, with sport scoring being one of its most common application scenarios. Existing methods are often divided into video-based and skeleton-based approaches [39]. In competitive sports, these methods can support automatic scoring, technical diagnosis, and feedback for gymnastics, diving, figure skating, martial arts, and sport-specific skill training.

Several challenges remain. Athletes often perform high-speed, large-amplitude movements under self-occlusion or multi-person occlusion, which can destabilize keypoint detection. Action structures and technical standards vary considerably across sports, making it difficult for general pose or action models to transfer directly to specialized scenarios. Errors in pose estimation can also propagate to downstream skeleton-based recognition and quality assessment. In addition, many studies rely on private datasets and customized algorithms, limiting reproducibility and practical adoption [35]. Future work should incorporate sport-specific knowledge, temporal constraints, multimodal sensing, and interpretable modeling to improve the stability and usefulness of pose estimation and action recognition in real-world sports settings.

Overall, pose estimation and action recognition support technical-skill analysis, training feedback, movement-quality assessment, and match-behavior understanding. Continued progress in markerless pose estimation, 3D human reconstruction, video Transformers, skeleton graph networks, and multimodal fusion will expand their role in sports analysis.

### 4.4. Spatiotemporal Modelling, Tactical Analysis, and Decision Support

After object detection, multi-object tracking, and pose estimation, sport performance analysis moves toward continuous process understanding and high-level decision support. Key events are embedded in temporal context, spatial structure, and competitive interactions; therefore, models must capture temporal dependencies among actions, spatial interactions among athletes, and game-state evolution.

In spatiotemporal modeling, event detection and action localization in sports videos often require long-term context. In football, events such as goals, shots, fouls, substitutions, and passes are strongly shaped by preceding and following situations, making single-frame or short-clip recognition insufficient. For football action spotting, context-aware loss functions have been proposed to improve localization by considering temporal segments before and after the event [40]. Other studies use temporally aware feature pooling to aggregate information from different phases around an event, strengthening the model’s ability to identify key moments [41]. These approaches show that sport event understanding depends not only on visual appearance but also on match rhythm, action duration, and contextual structure.

In team sports, game dynamics are characterized by constantly changing spatial relationships among players and the ball. Conventional temporal models can describe the evolution of individual targets or video segments, but they are less effective at explicitly representing interactions among players. Graph neural networks offer a natural solution: players and the ball can be modeled as nodes, while edges can encode spatial distance, passing relationships, defensive pressure, or relative positioning. Studies have shown that representing multi-agent movement trajectories as graphs is useful for analyzing spatiotemporal interaction and tactical behavior in team sports [42]. In football corner-kick scenarios, TacticAI constructs graph representations from player locations and geometric relationships to predict pass receivers, shot likelihood, and tactical adjustments, demonstrating the potential of graph neural networks for tactical analysis and decision support [6].

Spatiotemporal and graph-based methods can further support higher-level performance evaluation. Traditional performance metrics, such as goals, assists, distance covered, and pass success rate, often fail to capture the contextual value of an action. Recent work has used full-field player and ball-tracking data to estimate the potential attacking value of possession sequences. Expected possession value models, for example, evaluate how passes, carries, or shots contribute to scoring opportunities based on the evolving positions of players and the ball, shifting match analysis from outcome statistics toward process-oriented value assessment [43]. Such methods provide more fine-grained evidence for evaluating individual contributions, team coordination, and tactical choices.

From an application perspective, spatiotemporal modeling, tactical analysis, and decision support are shifting sport performance analysis from description toward explanation and intervention planning. In post-match review, models can help coaches identify spatial structures, movement patterns, and defensive vulnerabilities before key events. In real-time match analysis, trajectory prediction and tactical evaluation can support in-game decision-making. In long-term performance management, spatiotemporal metrics can help analyze player roles, team coordination, and tactical execution. Recent real-time football analysis has combined player tracking, player–ball interaction modeling, and graph convolutional networks to estimate inter-player distance, speed, total distance covered, and player–ball proximity, illustrating the shift from offline analysis toward real-time match intelligence [5].

Spatiotemporal modeling, graph neural networks, and decision-support methods form the high-level methodological layer of sport performance analysis. Future progress in this area will require deeper integration of complementary sensing modalities, improved interpretability, real-time capability, and adaptability across sports.

## 5. Deep Learning Methods for Sports Health Monitoring

Sports health monitoring focuses on identifying athletes’ internal load, fatigue responses, injury risk, and recovery status from continuously collected multisource data during training, competition, and rehabilitation. Unlike performance analysis, which emphasizes object detection, trajectory reconstruction, and tactical understanding, health monitoring prioritizes temporal continuity, individual variability, and early-warning capability. Model outputs are typically translated into training adjustments, clinical assessments, or return-to-play decisions. Deep learning approaches in this domain must therefore balance predictive accuracy, interpretability, real-time applicability, and practical utility in clinical and training contexts.

Given these requirements, health monitoring studies can be organized according to the clinical or training decisions they support. Table 3 summarizes the main deep learning tasks in this domain and clarifies how sensor inputs are translated into decision-relevant outputs.

Table 3 shows that health monitoring differs from performance analysis because it depends on longitudinal, individualized, and clinically meaningful labels. Model outputs should therefore be interpreted cautiously and linked to professional judgment, particularly for injury-risk alerts and return-to-play decisions.

### 5.1. Training Load and Fatigue Monitoring

Training-load and fatigue monitoring are foundational components of athlete health management. Training load can be divided into external and internal components. External load quantifies physical output, including distance, speed, acceleration, sprints, jumps, and impact forces, whereas internal load reflects physiological responses such as heart rate, heart-rate variability, respiration, blood oxygen, body temperature, and electromyography. Traditional monitoring relies on discrete testing, manual records, or single physiological metrics, which limits continuous assessment under real training conditions. Wearable inertial, positioning, and physiological sensors now provide objective data for load evaluation and fatigue recognition.

From a modeling perspective, these tasks are inherently multichannel time-series problems. CNNs extract local waveform and impact features; LSTM and GRU models capture temporal dependencies between load and physiological response; TCNs model multiscale temporal patterns through dilated convolutions; and Transformers with attention mechanisms highlight temporally or spatially critical features. Wang et al. integrated CNN, LSTM, and self-attention into a wearable- and cloud-based system to predict athlete health status, demonstrating the potential of combining multisource time series with deep learning [23]. Hu et al. extended this approach by integrating IoT sensors with TCN, BiLSTM, and attention for real-time monitoring and feedback in collegiate athletes, showing that hybrid temporal models are promising for live training evaluation [25].

For internal-load estimation, deep learning can infer physiological metrics that are difficult to measure continuously. Sheridan et al. compared LSTM, CNN, MLP, and XGBoost models for VO2 estimation using IMU, heart-rate, and respiration data in team sports and found that LSTM models trained on raw IMU data provided superior prediction of oxygen consumption during activity [21]. This finding indicates that inertial signals can reflect internal metabolic demands beyond external performance measures.

Fatigue recognition is more complex than load monitoring because fatigue is expressed through reduced movement stability, slower physiological recovery, altered muscle activation patterns, and increased output variability. Biró et al. used multivariate IMU time series to predict fatigue and fitness states, showing that acceleration, angular velocity, and posture signals provide objective markers of fatigue [44]. In sport-specific applications, muscle activation patterns and task characteristics are critical. Shen et al. combined ECG and EMG from smart garments with a TCN-LSTM framework for real-time fatigue detection in cycling, illustrating the value of integrating physiological signals with temporal deep learning models [45]. Hwang et al. further demonstrated that fusing sEMG and IMU data through a CNN-LSTM-Attention model improves fatigue-recognition stability by capturing both kinematic and muscular dynamics [46].

Deep learning is shifting training-load and fatigue monitoring from isolated metrics toward continuous, multisource, and individualized modeling. Challenges remain, including limited annotations in real training settings, substantial inter-individual variability, limited cross-sport generalization, and model interpretability. Future research should link load estimation, fatigue prediction, and individualized feedback more closely.

### 5.2. Injury Risk Prediction and Early Warning

Injury-risk prediction is a practical component of health monitoring that aims to identify potential risk factors during training or competition and provide early warnings rather than post-injury diagnosis. Injuries arise from complex interactions among training load, movement patterns, physical function, recovery, prior injuries, and game context. Machine learning and deep learning approaches emphasize multisource integration and dynamic risk modeling rather than reliance on fixed thresholds.

Data sources typically include external load, internal load, and functional assessments. External-load variables include distance, high-speed running, sprints, accelerations, and decelerations; internal-load variables include heart rate, perceived exertion, sleep quality, fatigue, and recovery; and functional assessments include jump performance, strength asymmetry, joint mobility, and lower-limb control. Haller et al. found that sleep quality, high-speed run acute-to-chronic load ratio, and countermovement jump height were key predictors in elite youth football injury monitoring [47]. Tsilimigkras et al. identified sprint counts, load scores, time in high heart-rate zones, total distance, and high-speed distance as significant predictors of non-contact muscle injuries in professional football [48]. These studies highlight the importance of short-term load fluctuations, cumulative training exposure, and deviations from individual baselines.

Frameworks that integrate subjective state, GPS data, competition statistics, and medically verified injuries are emerging. SoccerGuard, for example, integrates subjective health reports, training load, GPS tracking, match statistics, and injury records for professional female footballers and explores the effects of input/output window sizes and data-balancing strategies [49]. Machine learning methods such as random forests, SVM, gradient boosting, XGBoost, MLPs, and deep temporal networks have been applied to injury classification and risk prediction. Martins et al. showed that models built on four weeks of prior internal and external load data achieved strong AUC-PR and balanced accuracy in muscle injury prediction [9]. In endurance and triathlon settings, Rossi et al. emphasized that lifestyle factors, including sleep, stress, heart-rate variability, and recovery, interact with training load to influence injury risk [50].

Despite progress, several task-specific challenges remain: limited sample sizes, low injury incidence, inconsistent sport and injury definitions, insufficient external validation, and limited model interpretability [12]. Risk-prediction models should identify actionable causes of elevated risk, such as abrupt load increases, inadequate recovery, asymmetrical movement, or excessive high-intensity running, to support training adjustment, recovery intervention, and participation management.

The comparison between recent football studies also illustrates why AI conclusions should be treated as conditional on the measured variables rather than as universal injury mechanisms. Haller et al. used a broad monitoring battery that included external load, questionnaires, blood markers, neuromuscular tests, hamstring strength, and hip strength, but the prediction task still suffered from low precision and false-positive alerts [47]. By contrast, Tsilimigkras et al. focused on a smaller set of mechanical and physiological load variables over a 28-day pre-injury window and identified sprint counts, training-load scores, time in high heart-rate zones, total distance, and high-speed distance as important estimators [48]. These differences do not necessarily contradict one another; they show that the apparent risk factors learned by AI models depend on sensor selection, feature windows, label definitions, sport context, and baseline comparison strategy. Therefore, injury-risk models should report what was measured, what was not measured, and which factors, such as psychological stress, recovery behavior, prior injury, or position-specific demands, remain outside the model.

### 5.3. Rehabilitation Monitoring and Return-to-Play Decision Support

Rehabilitation monitoring and return-to-play decisions connect post-injury treatment, functional recovery, and competitive readiness. Traditional assessments, including strength testing, jump tests, range of motion, balance measures, and subjective scoring, capture recovery at discrete time points but cannot continuously track motion quality, load tolerance, or compensatory patterns. Machine learning and prognostic modeling are supporting more objective and individualized return-to-sport assessment [11,51].

In anterior cruciate ligament (ACL) reconstruction and similar contexts, return-to-play evaluation must assess not only rehabilitation completion but also readiness in terms of lower-limb strength, dynamic stability, symmetry, psychological preparedness, and sport-specific capability. Hwang et al. used post-surgery performance metrics at three months to predict 12-month return using random forests and gradient boosting, with jump height and functional scores as key inputs [11]. Systematic reviews confirm that strength, jump performance, physical function, and psychological readiness influence outcomes, whereas existing models often lack external validation and high-quality evidence [51]. This evidence indicates that return-to-play decisions should integrate functional recovery, performance, and psychological factors rather than relying solely on rehabilitation duration or fixed thresholds.

For this reason, rehabilitation and return-to-play systems should be framed as decision-support tools rather than autonomous decision-makers. Sensor-derived symmetry, strength, load tolerance, and movement-quality indicators can reveal important recovery trends, but they cannot fully capture pain, fear of reinjury, confidence, motivation, tactical role, or the clinical reasoning of physicians and physiotherapists. Psychological readiness is especially relevant because return-to-sport evidence after ACL reconstruction remains uncertain and is influenced by both physical and psychological prognostic factors [51]. Consequently, AI-based rehabilitation monitoring should combine sensor outputs with clinical examination, athlete-reported outcomes, sport-specific functional tests, and expert interpretation.

Wearables provide continuous monitoring during rehabilitation. IMUs, pressure insoles, and flexible sensors offer portability, low cost, and long-term tracking, capturing kinematic and kinetic changes during walking, running, stair tasks, jumps, landings, and sport-specific exercises. Chen et al. combined wearable IMUs with CNN-BiGRU-Attention models to estimate vertical ground reaction forces in ACL patients across functional tasks, demonstrating that deep learning can infer laboratory-measured metrics from wearable signals [52]. Tang et al. developed a smart garment with strain sensors and 1D ResNet-18 to classify movement quality in real time, detecting asymmetrical muscle activation and breathing coordination problems [53]. Digital rehabilitation platforms, including virtual-reality and interactive training systems, can support adherence monitoring, remote feedback, and longitudinal recovery management [54,55,56]. Pose estimation using RGB, depth, or mobile cameras extracts joint positions, joint angles, movement amplitude, center-of-mass displacement, and bilateral symmetry, providing fine-grained assessment of squats, balance tasks, gait, and other rehabilitation movements.

Deep learning is transforming rehabilitation monitoring and return-to-play assessment toward decision support for return-to-play readiness. Multisource data can reveal recovery trends, load tolerance, and compensatory risks, but future work must address limited sample sizes, rehabilitation-stage heterogeneity, cross-device consistency, external validation, and clinical interpretability.

### 5.4. Multimodal Fusion and Individualized Health Management

Multimodal fusion is important for individualized health management because no single modality can fully capture an athlete’s physiological state and load response. Building on the fusion mechanisms introduced in Section 3.5, this section focuses on how combined kinematic, physiological, spatial, and movement-quality signals support health-related decisions.

Health-monitoring models can apply these fusion strategies according to data availability and task requirements. Shen et al. integrated ECG, EMG, and workload data through a TCN-LSTM hybrid network for cycling-specific fatigue detection [45]. Hwang et al.’s CNN-LSTM-Attention model fused IMU and sEMG features for real-time multimodal fatigue-state recognition, illustrating the advantages of multimodal integration [46].

Multimodal fusion also enables individualized health management by accounting for fitness level, training history, injury history, and psychological state. Longitudinal data can model personal responses and guide adaptive training intensity or task difficulty. Li et al. reported that virtual-reality technology may improve knee function, walking function, gait function, and muscle strength after anterior cruciate ligament reconstruction, supporting the role of interactive digital rehabilitation rather than a specific multimodal sensing system [56]. Tang et al.’s smart garment similarly analyzes muscle symmetry and breathing coordination in real time [53].

Wearables, IoT platforms, and edge/cloud computing can facilitate closed-loop athlete monitoring and individualized training feedback by enabling data transmission, preprocessing, and timely feedback to coaches and athletes [25]. Despite progress, challenges remain, including temporal and spatial synchronization, missing or noisy signals, limited cross-individual generalization, and model interpretability. Future research should develop adaptive fusion, cross-modal attention, and explainable deep learning to enable actionable individualized monitoring.

## 6. Key Challenges and Future Directions

Although deep learning has shown considerable promise in sport performance analysis and athlete health monitoring, substantial gaps remain between current research and reliable real-world deployment. Sports environments involve high-speed motion, sport-specific variability, pronounced inter-individual differences, complex acquisition conditions, and limited annotations. These characteristics create persistent challenges in data quality, generalization, real-time deployment, interpretability, and practical translation.

To make the discussion easier to follow, the challenges are arranged from data-level issues to model-level issues and finally to translation-level issues. This order links limitations in sensor acquisition and annotation with generalization, multimodal fusion, real-time deployment, interpretability, ethical governance, and practical adoption.

### 6.1. Data Quality, Annotation Cost, and Standardization

High-quality data are the foundation of effective deep learning models, yet sports sensor data are often heterogeneous and unstable. Vision data are affected by occlusion, lighting variation, motion blur, and camera viewpoint. GPS and optical tracking data depend on sampling frequency, field conditions, and stable identity association. IMU and physiological signals are sensitive to sensor placement, attachment method, signal drift, motion artifacts, and individual variability. Despite their wide use in performance monitoring, injury prevention, and rehabilitation assessment, accuracy, cost, wearing comfort, privacy, and standardization continue to limit broader adoption [57]. Future work therefore requires not only better sensing hardware but also standardized protocols for data collection, variable definition, sampling frequency, synchronization, and label construction.

Insufficient annotation is another central limitation. Object detection, pose estimation, and action recognition require large amounts of frame-level, instance-level, or temporal annotations. Health-related tasks such as fatigue recognition, injury-risk prediction, and rehabilitation outcome modeling depend even more heavily on longitudinal monitoring and medically verified labels. Compared with general image recognition, key events in sports health monitoring, such as muscle injury, overtraining, and reinjury, are rare, leading to class imbalance and overfitting. Weakly supervised learning, semi-supervised learning, self-supervised pretraining, and active learning may help reduce annotation costs. At the same time, shared datasets across teams, seasons, and sports are needed to improve model stability and generalizability.

Based on these limitations, future studies should report a minimum set of contextual, sensing, modeling, and governance elements so that results can be compared, reproduced, and integrated into larger multimodal corpora. Table 4 summarizes recommended reporting elements for sensor-based sports AI studies, with particular relevance to injury-risk prediction, rehabilitation monitoring, and cross-sport generalization.

### 6.2. Limited Generalization Across Sports, Individuals, and Devices

Data distributions differ substantially across sports settings. Sports vary in movement structure, speed, physical contact, field conditions, and sensor configuration. Athletes also differ in body morphology, technical style, training level, fatigue response, and injury history. Models trained on a single team, sport, or device, therefore, often fail to transfer effectively to new contexts. Studies on multimodal wearable sensor-based human activity recognition have shown that multisensor systems can improve the representation of complex behaviors, but they also introduce sensor heterogeneity, modality-combination variability, cross-environment transfer problems, and missing-modality issues [58]. In sports applications, these problems are further amplified by competition settings, sport-specific actions, and individualized training needs.

Future studies should place greater emphasis on robustness and transferability. Domain adaptation, transfer learning, meta-learning, and individualized fine-tuning may help models adapt to different sports, devices, and athletes. Longitudinal models that capture individual baselines are also needed because the same indicator may have different meanings across athletes and competitive contexts.

Generalization should also be considered at the level of sport-specific and position-specific roles. In team sports, athletes occupying different positions may differ in morphology, repeated-sprint exposure, collision load, tactical responsibility, and recovery demands; for example, forwards and backs in rugby or defenders, midfielders, and forwards in football may not share the same risk profile or performance indicators. A model trained on aggregated athlete data may therefore learn an average pattern that is poorly calibrated for a specific position, sex, age group, or rehabilitation stage. Future models should incorporate hierarchical, domain-adaptive, or individualized designs that allow shared representations where appropriate while preserving sport-, position-, and athlete-specific parameters.

### 6.3. Technical Barriers in Multimodal Fusion and Real-Time Deployment

Multimodal fusion is a promising route to more robust sports intelligence, but practical implementation remains challenging. Sensing modalities differ in sampling frequency, temporal delay, coordinate system, noise profile, and missing-data pattern, so reliable synchronization, alignment, and integration are prerequisites for effective modeling. Reviews of multisensor data fusion for wearable health monitoring suggest that multisource fusion can improve reliability, accuracy, and robustness, but differences in data quality, acquisition context, and integration method remain major barriers [59].

Real-time deployment is equally important in sports. Feedback in training or competition often needs to be delivered with low latency, whereas complex deep learning models may be constrained by edge-device computation, power consumption, and network transmission. Recent studies have introduced edge computing and Internet of Things technologies into athlete monitoring systems, showing that combining sensing, edge processing, and deep learning can improve activity recognition, data processing, and real-time feedback [13]. Practical deployment still requires careful trade-offs among accuracy, inference speed, power consumption, device cost, and system stability. Lightweight networks, pruning, knowledge distillation, on-device inference, and cloud-edge collaboration will be essential for moving deep learning models from laboratory settings to fields, gyms, competitions, and rehabilitation environments.

### 6.4. Interpretability, Ethical Risks, and Practical Translation

In sport performance analysis and health monitoring, model outputs may influence training plans, competition selection, injury prevention, and return-to-play decisions. Interpretability and trustworthiness are therefore critical. A scoping review of explainable artificial intelligence in sports science found that XAI applications remain fragmented; SHAP-based approaches are common, whereas visualization, rule-based explanations, and user-centered explanation frameworks for practitioners remain underdeveloped [60]. Future research should move from nominal interpretability toward usable explanations linked to training load, movement patterns, recovery status, and tactical context.

For high-stakes applications, XAI should be regarded as a design requirement rather than a post hoc decoration. Explanations should indicate which variables, time windows, movement phases, or sensor streams contributed to a prediction, and they should be checked against domain knowledge from coaches, sports physicians, physiotherapists, and biomechanists. Current XAI research in sports science is still dominated by SHAP-based approaches, while visual, rule-based, and user-centered explanation methods remain limited [60]. Future studies should therefore validate explanations with practitioners and report whether the explanation is actionable for training adjustment, injury prevention, rehabilitation progression, or return-to-play decisions.

Sports sensor data often include sensitive physiological, behavioral, health, and training information. Their use raises ethical concerns related to privacy, informed consent, data ownership, algorithmic bias, and accountability. Fairness and bias, transparency and explainability, athlete privacy and data governance, and responsibility mechanisms are identified as core issues [61]. In health monitoring, biased models across sex, age, competitive level, or injury background may lead to unfair or unsafe decisions. Technical development should therefore be accompanied by data governance and ethical review frameworks that define what data are collected, how they are stored, who can access them, and how model feedback should be used. Intelligent monitoring systems should protect athlete health rather than become instruments of excessive surveillance or performance pressure.

Specific safeguards include obtaining ethics approval and informed consent before longitudinal monitoring, limiting data collection to task-relevant variables, de-identifying or pseudonymizing video, wearable, and clinical data, storing raw sensor streams in encrypted or access-controlled repositories, and restricting data access to authorized researchers, coaches, or clinical staff. For wearable and cloud-based monitoring systems, secure transmission, role-based access control, audit trails, and clear data-retention policies are particularly important because physiological signals and training histories may reveal sensitive health or performance information [23,24,61]. Participants should also be informed about how model outputs will be used, whether data may be shared, and how they can withdraw from monitoring. In injury-risk prediction and return-to-play settings, privacy protection should be coupled with human oversight so that model-generated alerts support, rather than replace, medical and coaching judgment.

### 6.5. Future Directions: From Intelligent Perception to Closed-Loop Decision-Making

Sensor-driven sports intelligence is moving from isolated perception and offline analysis toward multimodal fusion, real-time feedback, and individualized closed-loop management. Future research should strengthen large-scale, multisport, and multicenter datasets, especially for fatigue recognition, injury-risk prediction, and rehabilitation outcomes. Methodologically, the field should move from single-task optimization toward multitask learning that integrates perception, load assessment, fatigue monitoring, risk prediction, and rehabilitation feedback within shared representations.

Human-AI collaboration will also be central. Deep learning models should not replace coaches, physicians, or rehabilitation specialists but should provide timely, detailed, and interpretable information to support professional judgment. This is especially important in injury-risk prediction and return-to-play decisions, where model outputs must be considered alongside medical assessment, training goals, athlete-reported status, and competition context. Future systems should follow a closed-loop workflow of sensing, modeling, interpretation, warning, intervention, and outcome verification. In practical study design, human-AI collaboration should be operationalized through predefined review points, where model outputs are interpreted together with athlete-reported status, coaching observations, medical examination, and sport-specific constraints.

Large models, self-supervised learning, and multimodal foundation models may create new opportunities for sports sensor data analysis. Pretraining on large volumes of unlabeled video, trajectory, IMU, and physiological data could enable models to learn more general movement representations and reduce dependence on task-specific annotation. However, sports data are inherently sport-specific and individually variable. Foundation models will therefore need to be integrated with knowledge from biomechanics, training science, and sports medicine before they can be used reliably. The most valuable future sports intelligence systems will not simply be those with higher algorithmic accuracy but those that operate reliably in real environments, provide clear explanations, protect privacy, and support individualized training and health management. A useful near-term goal is not a single universal model for all athletes, but a common reporting and validation framework that allows models to be compared, adapted, and audited across sports, positions, devices, and populations.

## 7. Conclusions

This review examined recent progress in sensor-driven sport performance analysis and athlete health monitoring. Multisource sensing provides the data foundation for sports intelligence, while deep learning converts heterogeneous data into information for perception, movement understanding, tactical interpretation, load assessment, injury-risk prediction, and rehabilitation support. This field is moving from manual observation and post-event statistics toward continuous sensing, data-driven modeling, and real-time feedback. Taken together, the review shows that sensor-based sports intelligence should be interpreted through a unified chain from sensor choice to data representation, model design, application endpoint, and deployment constraint.

In sport performance analysis, deep learning models extract action features, interaction patterns, and match context from sensor data, allowing analysis to move beyond event identification toward explanation of movement patterns and tactical mechanisms. Graph neural networks, spatiotemporal modeling, and multi-object tracking provide technical routes for interpreting spatial relationships, tactical patterns, and decision-making processes in team sports.

In athlete health monitoring, deep learning processes multichannel temporal signals to identify workload changes, physiological responses, fatigue accumulation, injury risk, and functional recovery. Compared with traditional single-point testing or fixed-threshold assessment, these methods support more continuous, dynamic, and individualized health evaluation. Their reliability, however, depends on whether the selected variables adequately represent sport-specific load, individual baseline, psychological state, injury history, and professional clinical judgment.

Several challenges remain, including context-dependent data, limited high-quality annotations, sparse injury events, insufficient external validation, real-time deployment constraints, interpretability, privacy protection, and ethical governance. In high-stakes applications such as injury-risk prediction and return-to-play decisions, model outputs must be accurate, understandable, and actionable. AI outputs should be considered conditional on the measured data and modeling assumptions, particularly when applied to injury-risk prediction, rehabilitation progression, and return-to-play decisions.

Future sports intelligence should develop toward standardization, personalization, real-time operation, and closed-loop management. Cross-sport, cross-device, and cross-context datasets and evaluation standards are needed to improve comparability and reproducibility. Self-supervised learning, transfer learning, explainable artificial intelligence, edge computing, and adaptive multimodal fusion should be further developed to improve robustness and usability in real training, competition, and rehabilitation environments. This sensor-data-model-application perspective is intended to help future studies select appropriate measurement protocols, report comparable evidence, and translate deep learning outputs into responsible practice.

## Figures and Tables

**Table 1:** Summary of sensor modalities, data types, model architectures, applications, and limitations in sensor-based sport performance and health monitoring.

Sensor Modality	Representative Data Types	Typical Model Architectures	Main Applications	Key Limitations
Vision sensing	video frames, multi-view or depth images, pose/keypoint sequences	CNNs, 3D CNNs, video Transformers, pose-estimation networks, temporal attention models	Athlete, ball, and equipment detection; pose estimation; action recognition; event spotting; action quality assessment [3,15,16,17]	Occlusion, camera viewpoint changes, lighting variation, motion blur, high annotation cost, and privacy concerns
Trajectory, positioning, optical tracking	2D/3D coordinates, velocity, acceleration, distance, identity labels, player and ball trajectories	RNN/LSTM/GRU, TCN, Transformer, graph neural networks, spatiotemporal models	Movement pattern analysis, spatial occupation, tactical structure, player interaction, expected possession/value, decision support [18,19]	Tracking errors, calibration dependence, sampling heterogeneity, identity switches, and limited physiological context
Wearable inertial sensing	Accelerometer, gyroscope, magnetometer/IMU, orientation, impacts, step frequency, segmental motion	1D-CNN, LSTM/GRU, TCN, temporal Transformer, attention-based sequence models	Training load estimation, action-cycle detection, fatigue indicators, rehabilitation movement assessment, non-invasive internal load estimation [20,21]	Sensor placement effects, drift and noise, attachment instability, limited spatial semantics, and inter-athlete variability
Physiological and biosignal sensing	Heart rate, HRV, ECG, EMG/sEMG, SpO2, respiration, skin temperature, sweat-related indicators	CNN-LSTM, GRU, TCN, attention mechanisms, sequence-to-state prediction models	Internal load estimation, fatigue and recovery monitoring, health-state recognition, early warning, individualized training adjustment [22,23]	Motion artifacts, sweating and skin-contact instability, individual baseline differences, and context-dependent interpretation
Multimodal fusion	Synchronized video, trajectories, IMU/GPS, physiological signals, smart garments, rehabilitation data	Feature-level and decision-level fusion, cross-modal attention, multimodal Transformers, graph-fusion models, ensemble models	Robust action recognition, load and fatigue modelling, injury risk prediction, rehabilitation feedback, personalized health management [7,21,24]	Temporal synchronization, missing modalities, feature imbalance, data governance, computational cost, and deployment complexity

**Table 2:** Deep learning methods for sport performance analysis: inputs, model families, outputs, representative evidence, and limitations.

Performance-Analysis Task	Sensor/Data Inputs	Representative Deep Learning Methods	Outputs and Representative Evidence	Key Limitations
Athlete, ball, and equipment detection	Images and video frames from broadcast, fixed-camera, or training systems	YOLO-series detectors, CNN detectors, lightweight detection networks	Object localization, ball/player detection, and normative movement detection; representative studies include YOLOv4, YOLO reviews, DeepSportLab, SCB-YOLOv5, and YOLOv7-based soccer-ball tracking [26,27,28,29,30]	Small and fast-moving objects, player overlap, similar uniforms, background clutter, and real-time inference constraints
Multi-object tracking and trajectory reconstruction	Detected players/balls, video sequences, identity labels, field calibration, and tracking trajectories	DeepSORT-type association, domain-specific MOT, motion-vector augmented segmentation, re-identification and matching models	Identity preservation, pitch localization, speed, distance, formation and spatial metrics; representative studies include soccer tracking surveys, MV-Soccer, SportSORT, and FootyVision [31,32,33,34]	Camera motion, long occlusion, identity switches, calibration error, fragmented trajectories, and sport-specific tracking assumptions
Pose estimation and action recognition	RGB/depth video, skeleton sequences, optical flow, pose keypoints, and action clips	2D/3D pose networks, skeleton-based models, RGB-pose fusion, video action recognition networks	Technical feedback, action category recognition, phase segmentation, and training assistance; representative studies include pose-estimation reviews, AI Coach, team-sport action-recognition surveys, and Gate–Shift–Pose [35,36,37,38]	Self-occlusion, high-speed motion, sport-specific movement standards, private datasets, and error propagation from pose estimation
Action quality assessment and event spotting	Fine-grained video clips, procedure annotations, match videos, temporal labels, and scoring data	Temporal attention, context-aware losses, temporally aware pooling, video/skeleton-based quality-assessment models	Movement quality scores, action phase analysis, and temporal event localization; representative evidence includes FineDiving, action-quality assessment reviews, and SoccerNet action-spotting methods [16,39,40,41]	Subjective scoring criteria, long temporal context, annotation ambiguity, and difficulty transferring models across sports
Spatiotemporal modelling, tactical analysis, and decision support	Player–ball trajectories, possession sequences, spatial relations, interaction graphs, and game-state variables	Graph neural networks, spatiotemporal graphs, expected possession/value models, geometric deep learning	Tactical pattern analysis, pass/shot value estimation, player–ball interaction analysis, and decision support; representative studies include real-time player–ball GCN analysis, TacticAI, graph-based sport-data analysis, and expected possession value modelling [5,6,42,43]	Limited interpretability, transfer across teams and competitions, real-time computational cost, and dependence on accurate tracking data

**Table 3:** Deep learning methods for sports health monitoring: sensor inputs, model types, decision outputs, representative evidence, and limitations.

Health-Monitoring Objective	Sensor/Data Sources	Representative Model Types	Outputs and Decision Use	Key Limitations
Training load and internal-load estimation	IMU, GPS/local positioning, heart rate, respiration, session workload, and training context	LSTM/GRU, CNN, TCN, attention models, MLP/XGBoost baselines	External/internal load estimation, VO2 prediction, real-time training feedback, and health-state modelling [21,23,25]	Small samples, sport-specific movement demands, individual baselines, limited ground truth, and deployment constraints
Fatigue and state recognition	IMU time series, ECG, EMG/sEMG, smart garments, workload data, and recovery indicators	TCN-LSTM, CNN-LSTM-Attention, temporal Transformers, multichannel sequence models	Fatigue or fitness-state recognition, recovery monitoring, and real-time alerts [44,45,46]	Subjective or noisy fatigue labels, inter-athlete variation, signal artifacts, and limited cross-sport generalization
Injury risk prediction and early warning	External load, GPS variables, heart rate/RPE, sleep, recovery, jump performance, strength asymmetry, injury history	Random forests, SVM, gradient boosting, XGBoost, MLPs, temporal models, explainable ML	Risk classification, early warning, and identification of modifiable risk factors [9,10,47,48,49,50]	Rare injury events, class imbalance, inconsistent injury definitions, insufficient external validation, and limited interpretability
Return-to-play decision support	Early physical performance tests, strength and jump metrics, functional scores, psychological readiness, and longitudinal recovery data	Random forests, gradient boosting, prognostic models, explainable ML	Return-to-sport probability, readiness estimation, and risk-informed participation decisions [11,51]	Limited evidence quality, insufficient external validation, ethical accountability, and need for medical and coaching judgment
Rehabilitation monitoring	IMU, pressure/force proxies, RGB/depth pose, smart garments, digital rehabilitation platforms, and functional tasks	CNN-BiGRU-Attention, 1D-ResNet, pose-estimation models, digital monitoring systems	Vertical ground-reaction-force estimation, symmetry and movement-quality assessment, adherence monitoring, and home-based feedback [11,52,53,54,55]	Rehabilitation-stage heterogeneity, clinical-threshold uncertainty, device comparability, limited longitudinal validation, and privacy concerns
Personalized closed-loop health management	Multimodal longitudinal data, IoT/wearables, smart garments, VR/digital rehabilitation, and cloud/edge systems	Multimodal fusion, cross-modal attention, adaptive models, edge/cloud deep learning	Individualized feedback, intervention adjustment, long-term monitoring, and digital rehabilitation support [25,56,57,58,59]	Synchronization, missing data, algorithmic bias, privacy and data governance, user adherence, and practical usability

**Table 4:** Recommended reporting elements for sensor-based sports AI studies.

Element	Minimum Information	Purpose
Sport and task context	Sport, competitive level, sex/age group, playing position or role, training/competition setting, and task objective.	Clarifies whether findings can transfer across sports, positions, and athlete groups.
Participant and clinical profile	Anthropometrics, training history, injury history, rehabilitation stage, psychological readiness, and relevant medical constraints.	Supports individualized interpretation rather than treating athletes as interchangeable data points.
Sensor and acquisition protocol	Device type, placement, sampling rate, calibration, attachment method, synchronization, missing-data handling, and environmental conditions.	Improves reproducibility and cross-study comparability of wearable, vision, trajectory, and physiological data.
Variables and labels	External-load, internal-load, biomechanical, physiological, psychological, and recovery variables; injury, fatigue, and return-to-play label definitions.	Reduces ambiguity in model inputs and outcomes, especially for injury-risk and rehabilitation models.
Modeling and validation	Model series, feature window, class imbalance strategy, training/test split, external validation, uncertainty, and subgroup or position-specific performance.	Limits overinterpretation of models trained on small or context-specific datasets.
Interpretation and implementation	XAI method, clinically or practically interpretable outputs, expert review, action thresholds, and intended decision workflow.	Connects AI outputs with coaches, physicians, physiotherapists, and athlete-centered decisions.
Ethics and governance	Consent, data minimization, privacy protection, access control, storage policy, data-sharing rules, and withdrawal procedures.	Protects athletes while enabling responsible data reuse and multimodal fusion.

## Data Availability

No new data were created or analyzed in this study. Data sharing is not applicable to this article.

## Abbreviations

AI	Artificial intelligence
CNN	Convolutional neural network
ECG	Electrocardiography
EMG	Electromyography
GNN	Graph neural network
GPS	Global positioning system
GRU	Gated recurrent unit
HRV	Heart-rate variability
IMU	Inertial measurement unit
IoT	Internet of Things
LSTM	Long short-term memory
MLP	Multilayer perceptron
RNN	Recurrent neural network
sEMG	Surface electromyography
SHAP	SHapley Additive exPlanations
SpO2	Peripheral oxygen saturation
SVM	Support vector machine
TCN	Temporal convolutional network
VO2	Oxygen uptake
XAI	Explainable artificial intelligence
YOLO	You Only Look Once
